# Altered brain network dynamics in youths with autism spectrum disorder

**DOI:** 10.1007/s00221-016-4737-y

**Published:** 2016-07-27

**Authors:** Evie Malaia, Erik Bates, Benjamin Seitzman, Katherine Coppess

**Affiliations:** 1 Netherlands Institute for Advanced Study, Meijboomlaan 1, Uil 11, 2242 PR Wassenaar, The Netherlands; 2 Stanford University, Stanford, CA USA; 3 Washington University in St. Louis, St. Louis, MO USA; 4 University of Michigan, Ann Arbor, MI USA

**Keywords:** Autism spectrum, Development, Network analysis, Resting state

## Abstract

The heterogeneity of behavioral manifestation of autism spectrum disorders (ASDs) requires a model which incorporates understanding of dynamic differences in neural processing between ASD and typically developing (TD) populations. We use network approach to characterization of spatiotemporal dynamics of EEG data in TD and ASD youths. EEG recorded during both wakeful rest (resting state) and a social–visual task was analyzed using cross-correlation analysis of the 32-channel time series to produce weighted, undirected graphs corresponding to functional brain networks. The stability of these networks was assessed by novel use of the L^1^-norm for matrix entries (edit distance). There were a significantly larger number of stable networks observed in the resting condition compared to the task condition in both populations. In resting state, stable networks persisted for a significantly longer time in children with ASD than in TD children; networks in ASD children also had larger diameter, indicative of long-range connectivity. The resulting analysis combines key features of microstate and network analyses of EEG.

## Introduction

Autism spectrum disorders are neurodevelopmental disorders characterized by behavioral impairments in social interaction, communication, and behavioral flexibility, as well as pathophysiological connectivity abnormalities in brain networks (Sun et al. 2012; Voineagu et al. 2011; Geschwind and Levitt 2007; Uhlhaas and Singer 2006). Understanding neural bases of autism requires analysis methods that capture dynamic differences in processing across heterogeneous participants and groups, including spatiotemporal balance between specialized and integrated processing in the brain.

Quantitative analyses of EEG in ASD indicate that oscillatory activity differs between typically developing (TD) and ASD populations, and several specific biomarkers have been proposed as possible diagnostic tools (e.g., band-specific power, coherence, and functional connectivity within specific bands; Barttfeld et al. 2011; Peters et al. 2013; Cherkassky et al. 2006; Kennedy and Courchesne 2008; Uddin et al. 2013). Understanding neurobiological processes that underlie brain connectivity abnormalities in ASD as a developmental disorder is critical for diagnostics and development of early targeted interventions. Spatial network analyses of functional connectivity using fMRI suggest that network development in ASD participants has a specific developmental profile: namely comparative overconnectivity in late childhood in ASD patients’ changes to underconnectivity in adolescence (Supekar et al. 2013). The mechanism behind these developmental changes is affected at the temporal scale of oscillatory activity of the brain, in which hierarchical networks at multiple frequencies continually arise and dissipate. In typically developing (TD) populations, developmental maturation in oscillatory coherence is instrumental to successful development of “small-world” networks. As the phase locking between low- and high-frequency neural activity increases toward late adolescence in TD populations, it is followed by a transitory decrease during transition to adulthood, which marks the developmental shift from long-range diffuse networks to local networks connected through central hubs (Chu et al. 2012). Accurate characterization of altered developmental trajectories of brain networks in autism, and resolution of the conflicting findings of hypo- and hyper-connectivity in ASD participants, requires developmental cross-frequency oscillatory network analysis.

The functional dynamics of active brain networks may be captured via several neuroimaging modalities, including electroencephalography (EEG). EEG is a noninvasive method of recording the brain’s electrical activity from the surface of the human scalp. The high temporal resolution of EEG recordings allows for the examination of brain dynamics on the millisecond timescale. Brain activity is recorded as potential changes in time, thereby generating a number of time series that each correspond to one measurement site. EEG time series may be used to understand functional networks of the brain and, in particular, how those networks evolve over time. One topic of interest in the neuroscience community is if and when such networks remain quasi-stable (Betzel et al. 2012; Koenig et al. 2002; Lehmann and Skrandies 1980). The primary goal of this work was to investigate the temporal dimension of disordered connectivity hypothesis in autism (Rippon et al. 2007), which characterized ASD as based on reductions in global connectivity and increase in local connectivity of neural networks. We use the term “functional connectivity” to describe the temporal relationship between sensor signals, measured by cross-correlating time series. Connectivity analyses of EEG data allow to bypass the problem of source localization (“inverse problem”) by developing mathematical models of networked activity based on sensor sites. Here, we characterize spatiotemporal network dynamics in EEG of typically developing (TD) and ASD youths.

## Methods

### Participants

Fourteen individuals (age range 8–15) with diagnoses of Asperger syndrome or high-functioning ASD and 14 healthy, typically developing (TD) age and gender-matched subjects recruited by advertisements among the schools of Arlington School District, TX, participated in the study. The participant numbers here are sufficient for hypothesis testing: Significant result, based on a small sample size, indicates the effect that is larger than the equivalent result with a large sample. Further, outliers decrease the type I error of parametric tests, such that significant result is even less likely to be a false positive (Friston 2012). Participants’ parents were asked to evaluate their children’s communicative abilities using the Pragmatic Language Observation Scale (Newcomer and Hammill 2009). Participants scoring at or above average (90 and above) on the Pragmatic Language Observation Scale were assigned to the control (TD) group; participants with scores lower than one standard deviation below average (84 and below) were assigned to the ASD group.

The study was approved and conducted in accordance with the ethical standards of the University of Texas at Arlington Institutional Review Board, and the ethical standards prescribed in the 1964 Declaration of Helsinki and its later amendments. All parents provided their written, informed consent, and children provided written, informed assent prior to their inclusion in the study.

### EEG recording and processing

Scalp EEG was recorded from 32 Ag/AgCl electrodes mounted in an electrode cap (Wavegard, ANT Inc.) with an average mastoid reference. Electrodes were positioned according to the standard 10–20 system. A pair of bipolar electrodes was used to record vertical eye movements. Electrode impedances were maintained below 10 k during recording. The EEG analog signal was digitized at a 512-Hz sample rate.

### Procedures

During the EEG session, the participants were seated comfortably in a sound-attenuating booth with their eyes approximately 80 cm from a computer screen. The participants were asked to keep their eyes on the screen and to decide as accurately and as quickly as possible whether the stimulus photograph expressed fear or anger. When the face–body compound stimuli were presented, participants were told to judge the expression of the face. Stimuli were presented for 1000 ms, followed by a black screen for 2000 ms. The hand assigned to fear/anger response was balanced among participants. The testing started with a short training session to acquaint participants with procedures and task expectations of the experiment. Examples from all four stimulus categories were included in the training. The study consisted of four blocks: two separate blocks when participants viewed isolated faces and bodies and two blocks with compound stimuli. Each block/category consisted of 40 stimulus trials, for a total of 160 trials. The order of block presentation varied, such that half of the participants viewed a face- or body-only block first, and half viewed a block with composite stimuli first. Of those who first saw a control block, half started with the isolated faces block, and half started with the isolated bodies block. For the purposes of this study, the EEG data from only one event type (anger in the body, no face shown) were used. Hereafter, this data will be referred to as “event-related” data.

### Signal processing

EEG data were analyzed using ASA 4.6 (ANT, Inc.). The continuous resting EEG data were shortened to one 500-ms-long epoch, and no other processing was performed. The continuous event-related EEG data were segmented into epochs of 500 ms consisting of data from 100 ms pre-stimulus onset and from 400 ms post-stimulus offset. Time points in the filtered data at which the absolute amplitude of the EEG exceeded 150 µV were marked as EEG artifacts or blink artifacts. Trials containing EEG artifacts were rejected from further analyses, as were trials containing incorrect behavioral responses. Averages were baseline corrected using the 100 ms pre-stimulus portion of the epoch. Ten TD children and ten children with ASD had EEG data in both conditions satisfying the rejection criteria.

We then represented each of the *n* = 32 nodes of EEG data by a real-values raw temporal signal *x*_*n*_(*t*), which was parsed into temporal windows, or epochs. This resulted 32 signals, $x_{{n,{\text{HF}}}} (\tau )$ for each epoch. A simple 1:1 (bivariate) cross-covariance analysis was conducted to obtain the correlation between every combination of nodes $\left[ {\hat{\gamma }_{ij} (h)} \right]$ for an optimal time lag (*h*), where *i* and *j* each index through the 1–32 nodes. The optimal time lag (*h*) leading to a maximum correlation value was selected to represent the magnitude of correlation within that epoch for each node pairing. High correlation was equated to an active network. Once matrix of internode correlations was obtained for each epoch within the full data series, the stability of the networks was analyzed by calculating change (edit distance) from one epoch to the next:

1 $${\text{dist}}(S_{1} ,S_{2} ) = \sum\limits_{i = 1}^{n} {\sum\limits_{j = 1}^{n} {(S_{1,ij} - S_{2,ij} )} }$$

Active networks within one epoch (*S*_1_) with a minimal change to the next (*S*_2_) were identified as stable over that time period. The changes from one connectivity matrix to the next matrix (network transitions) with an edit distance more than two standard deviations below the mean of the null model were considered stable. We then generated two separate null models for every subject. The first null model was generated by randomizing every matrix (i.e., graph) for every subject. This randomization was executed by use of the *null model und sign function* (with the default settings) in the Brain Connectivity Toolbox (Rubinov and Sporns 2010), which reassigns edge weights while preserving the weight, degree, and strength distributions of each graph.^1^ The second null model was generated by applying randomized temporal shifts to all recording channels (electrodes), in order to distort the network structure of individual data. Each time series contained 512 data points for each second of recording; 35-s epochs were used. The randomization was performed by deleting a random amount of time between 0 and 5 s from the start of time series. That is, instead of starting at the first data point, the randomized time series was taken to begin at the *n*th data coordinate, where *n* was a random integer uniformly chosen between 1 and 2560. These randomized time series were then trimmed to 30 s in duration so that each original time series yielded the same length of randomized time series, regardless of the randomized starting time.

**Fig. 1:**
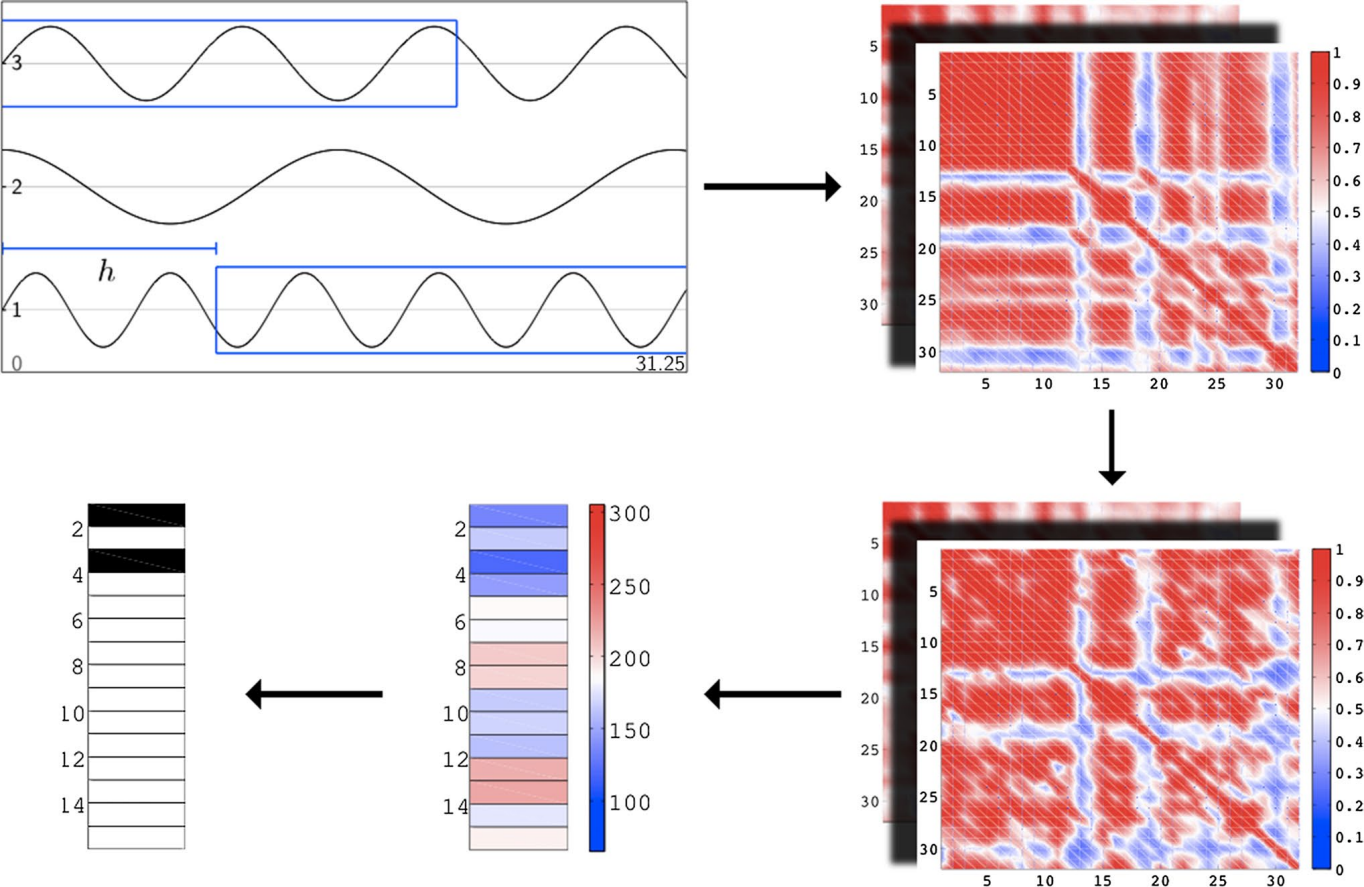
Each subject’s processed EEG signal leads to 16 functional matrices, one for each epoch, when cross-correlation is computed for each pair of nodes and all possible lags. The *red* cells seen in the top matrix indicate pairs of nodes for which there existed a lag producing a high cross-correlation value. All such matrices are randomized, and the edit distances (Betzel et al. 2012) between them are established the null model. Then, the null model was used to detect stable network transitions between the original matrices by thresholding edit distance vectors against the two standard deviations cutoff. Stable transitions are denoted by *black* cells. The example shown here had stable transitions between epochs 1 and 2 and between epochs 3 and 4, as suggested by their dark blue coloring in the edit distance vector (color figure online)

Two mean edit distances of the null model were calculated, one for each task condition. The mean for resting state network transitions was computed using both populations’ randomized resting matrices and that for event-related transitions using both populations’ randomized event-related matrices. For each null model, the stability threshold was established as described above for the corresponding condition. Further repeating this process through all epochs within the data set allowed for calculation of active and stable networks (Fig. 1). For every participant, an averaged stable network was computed for each period of quasi-stability, with each of two null models. Several network measures were calculated for all averaged stable networks by use of the Brain Connectivity Toolbox, including diameter, radius, characteristic path length, transitivity, maximum modularity, and global efficiency (Rubinov and Sporns 2010).

## Results

### Results using null model und sign function to generate the null model

**Fig. 2:**
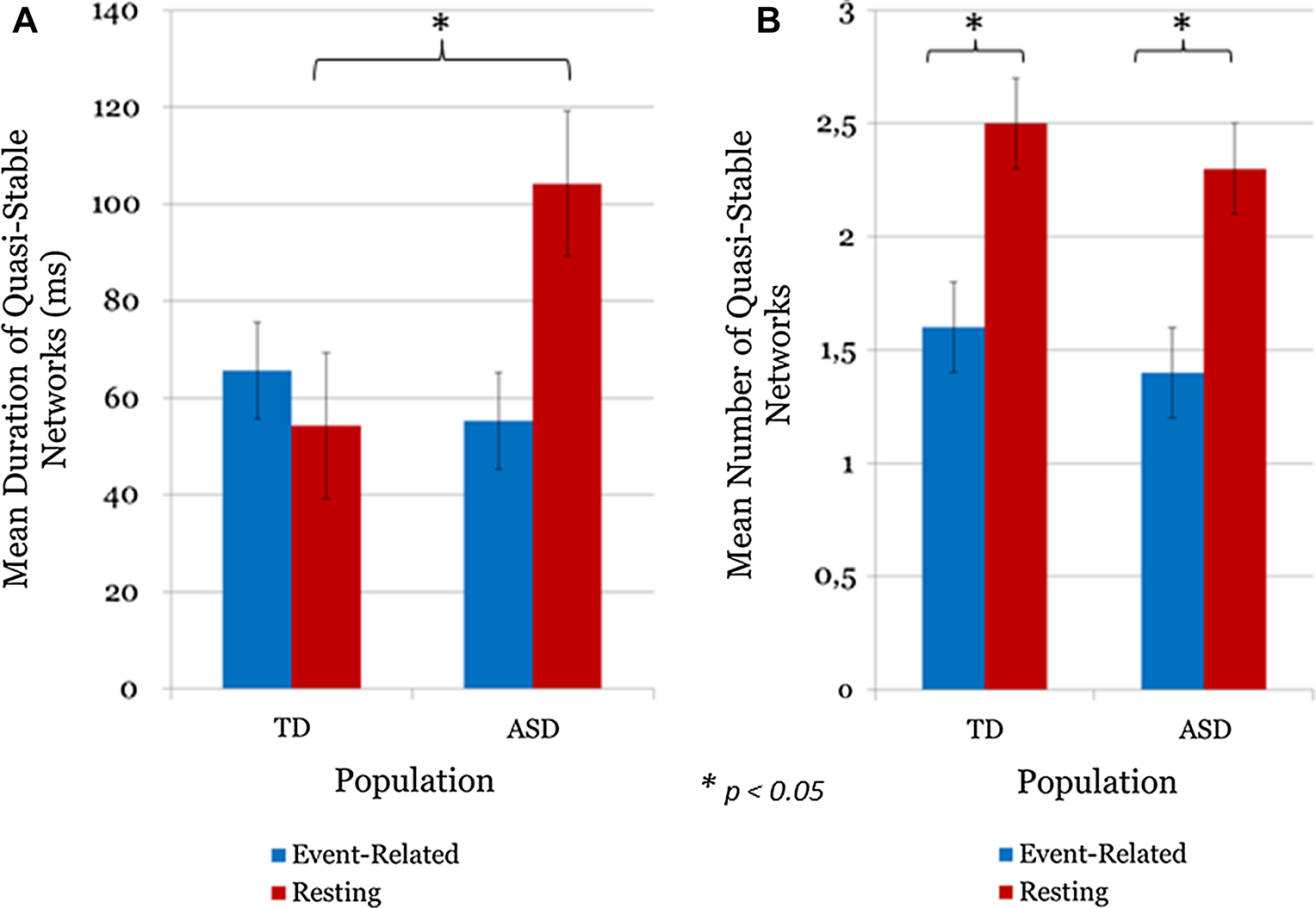
**a** Interaction between mean duration and population on the two conditions (*p* < 0.034) is driven by the resting condition that had a longer mean duration in children with ASD (see **a**). **b** Main effect of mean number of quasi-stable networks in the two conditions (*p* < 0.046), such that children with ASD had longer stable networks in the resting condition (see **b**)

A repeated measures ANOVA was performed for the average number of stable network transitions and the mean duration of stable network transitions with a between-subjects factor of population (TD vs. ASD). Children with ASD showed longer mean duration of quasi-stable networks in the resting condition (interaction between mean network duration and population, *p* < 0.034, Fig. 2a). The number of quasi-stable networks in the resting conditions was higher in both groups (*p* < 0.046, Fig. 2b), as expected, since rotation among networks in the resting state is not restricted by task demands.

**Fig. 3:**
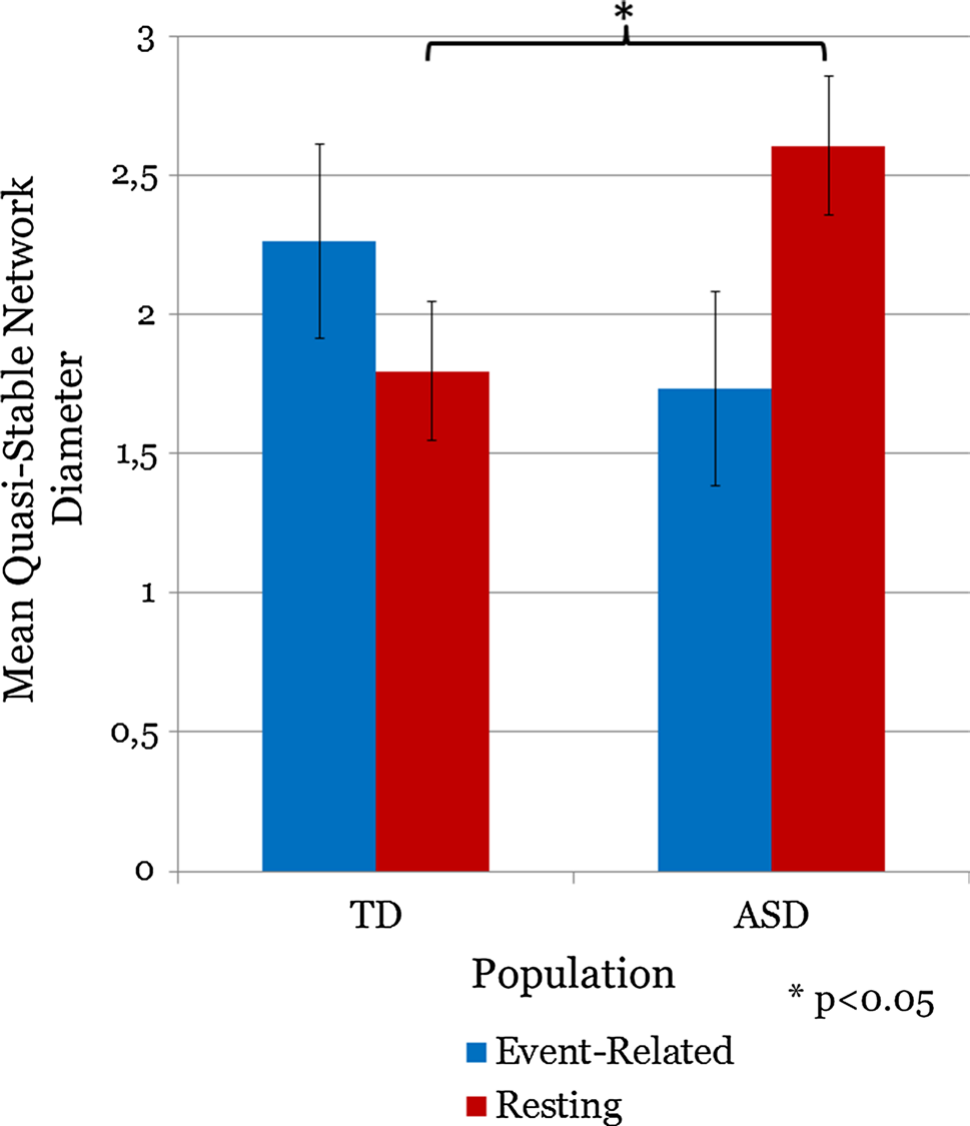
Interaction between mean network diameter and population type (TD, ASD) in the event-related versus resting state indicates that children with ASD have a larger mean network diameter in the resting state (*p* < 0.032)

Children with ASD had a larger mean network diameter in the resting condition (interaction significant between mean network diameter and population type (TD, ASD) in event-related versus resting state conditions, *p* < 0.032, Fig. 3).

### Results using randomized temporal shifts to generate the null model

A repeated measures ANOVA was performed for each of the network measures, with a between-subjects factor of population (TD vs. ASD). For the network radius, condition (event vs. rest) emerged as a significant factor (*p* < 0.019). A step-down analysis using independent samples *t* test indicated that for event-related networks, TD group had significantly larger mean network radius (*p* < 0.014), as compared to ASD group.

### Results using alternative distance metrics

The above results were obtained by use of the edit distance metric (Betzel et al. 2012). We also conducted additional analyses using *l* − 2 and normalized cosine similarity metrics (Bullmore and Sporns 2009). For those metrics, no stable microstates were detected in the data. Repeated measures ANOVAs for the rest of computed network measures for the event-related and resting data with the same between-subjects factor were not significant (*p* > 0.05). This suggests these metrics are not sensitive enough to detect microstate differences that are identified using *l* − 1 metric.

## Discussion

The results from this study support the existence of quasi-stable functional brain networks that are continually revisited while the brain is at rest (Betzel et al. 2012; Koenig et al. 2002; Lehmann and Skrandies 1980). At rest, the brain cycles through a larger variety of quasi-stable functional brain networks, as compared the brain that is responding to a stimulus,^2^ as confirmed by the results in this study concerning the number of stable network transitions. The observation that stable networks during resting state endure for a longer period of time in children with ASD suggest a direction for further research into the nature of brain abnormalities caused by ASD. Regardless of the type of null model used (matrix randomization vs. temporal shift), there were significant differences related to path lengths^3^ measures observed between TD and ASD participants. Use of time-shift null model indicated the larger mean network radius (minimum node eccentricity) in task-related condition in TD group, which is indicative of more correlated neural activity in spatially distributed networks in TD participants during task. Use of matrix randomization null model indicated that in the resting state, ASD participants had a larger network diameter (maximum node eccentricity): i.e., during rest, these participants have shown weakly correlated activity in more spatially distributed networks. The key issue in both cases appears to be cohesiveness of neural network activity, or effective cross-network integration of activity of multiple distributed networks, which is higher in TD participants, especially under cognitive load. These group differences in both resting and cognitive load states are indicative of altered developmental trajectory in the ASD population: The developmental shift from long-range diffuse networks to local networks connected through central hubs appears to be more drastic in ASD participants, as adults with ASD have been found to have locally overconnected networks (Peters et al. 2013).

A limitation of the study is that we accepted the school’s IQ measures available for the students, in lieu of conducting an IQ test for each participant. Future studies should assess IQ when the study is conducted so that a single standard assessment method is used.

The fact that network-level differences in parameters between ASD and TD populations are detected using the *l* − 1, but not the *l*− 2 metrics suggests that the difference between stable and unstable network transitions is more related to small changes in correlation between consecutive epochs, as opposed to large changes. Future studies should probe the effects of alternative metrics, such as the elastic net regularization, which is the sum of the *l* − 1 and *l* − 2 metrics.

Overall, parameters of spatiotemporal network analyses, such as used in the present study, can be helpful for early diagnostics of neurodevelopmental disorders. Of importance are also the noninvasive nature of data collection method (EEG, which has been used in young children, including neonates) and the possibility of using resting state data (for example, from very young children, for whom task-based metrics are not available). Analyses such as the present allow for an investigation of rich, high-dimensional neural oscillatory dynamics in ASD and other specific populations over the developmental trajectory, helping trace the cascading effects of early neural processing deficits on later brain and behavioral development.

## Footnotes

1 An alternative construction of the null models randomizes only one subject’s functional connectivity matrices, creating a null model specific to each subject for each condition. This approach, however, does not accurately capture variability between subjects because individualized null models are entirely determined by the randomization of only one subject’s functional connectivity matrices. Instead, the method described above offers a global perspective of typical edit distances between independent and pseudorandom networks, thereby lending a constant threshold with which to compare the network transitions of all subjects in a given condition. However, the configuration of edge weights in the null model can result in a connectivity matrix that is mathematically unrealizable. We thus used an alternative method by applying a temporal shift method of null model generation, in order to better understand the data.

2 In the long term, however, task-related network selection does affect resting state network connectivity (see Malaia et al. 2014).

3 The network radius and the network diameter are two measures related to node eccentricity: shortest path length between a node and any other node. The radius is the measure of minimum node eccentricity, and the diameter is the measure of maximum eccentricity.
